# Synthesis of a Flaky CeO_2_ with Nanocrystals Used for Polishing

**DOI:** 10.3390/ma17122859

**Published:** 2024-06-12

**Authors:** Yiming Zhang, Li Gou

**Affiliations:** College of Biomedical Engineering, Sichuan University, Chengdu 610065, China; zhangyiming1216@gmail.com

**Keywords:** cerium, crystal growth, materials science, morphology

## Abstract

It is important to adapt the morphology of CeO_2_ to different applications. A novel flaky CeO_2_ with nanocrystals was successfully synthesized using the ordinal precipitation method and calcination. The size of the flaky CeO_2_ was about 10 μm, and the nanocrystals were about 100 nm. Under the action of the precipitant NH_4_HCO_3_, Ce^3+^ nucleated in large quantities. The nanosized crystals gathered into flakes driven by the surface energy. As the calcination temperature increased, the grains grew slowly by mass transfer due to the slow diffusion of reactants. By adding AlOOH to the starting material, the Al^3+^ doped into the CeO_2_ increased the content of Ce^3+^ in the CeO_2_, which improved the chemical activity of the CeO_2_. When the starting material’s Al:Ce ratio was 5:1, the Ce^3+^ increased to 31.11% in the CeO_2_, which provided good application potential in the polishing field. After polishing by the slurry of flaky CeO_2_ for 1 h, the SiC surface roughness reduced from 464 nm to 11 nm.

## 1. Introduction

The crystal structure of cerium oxide is an open fluorite structure consisting of Ce^4+^ arranged in a face-centered cubic arrangement and O^2−^ occupying tetrahedral positions. Due to the intrinsic characteristics of atomic hybridization to form a cubic fluorite lattice structure, cerium oxide has intrinsic Ce^3+^ and its content is easily affected by doping and the formation of oxygen vacancies [1]. Such a structure makes it easy to form oxygen vacancies and perform element doping, and it maintains the cubic structure of fluorite with good chemical stability [2]. Due to its excellent properties, cerium oxide is currently used in a wide range of fields, such as CO_2_ conversion catalysis [3], photocatalysis [4], ultraviolet absorption [5], biomaterials [6], thermal coatings [7] and polishing abrasives [8].

The issue of how to prepare specific sizes and morphologies of cerium oxide is of great significance when it comes to adapting it to different applications. At present, researchers have successfully prepared CeO_2_ from different dimensionalities, including nanosphere-like and nanowire-shaped, nanoflakes, and nanocubes through different preparation methods. Among them, two-dimensional cerium oxide materials have huge application potential because of their high specific surface area and high anisotropy. However, they are difficult to synthesize due to a lack of intrinsic driving force during growth. Thus, only several reports discussed the synthesis of two-dimensional cerium oxide. C.H. Lu et al. prepared rhombic cerium hydroxycarbonate through the hydrothermal method [9]. L. Chen et al. synthesized larger-sized flaky cerium oxide by preparing precursors from the solution with a low concentration and pyrolysis [10]. J. Gong et al. controlled the morphology of the precursor CeOHCO_3_ through the hydrothermal method and synthesized equilateral triangular nanosheets of CeO_2_ with excellent optical properties [11].

In this paper, a novel flaky CeO_2_ composed of nanocrystals was prepared and the nanocrystal formation mechanism was discussed. The potential application of this material was explored in the field of polishing. Since aluminum is difficult to replace in cerium oxide crystals during the preparation process, a small amount of the Al element was doped into the lattice of the CeO_2_ to enhance its performance in the polishing process. In contrast, the Al^3+^, which did not dope in the CeO_2_, transformed into Al_2_O_3_ after precipitation and calcination. The Al_2_O_3_ was mixed with CeO_2_ to improve the mechanical strength.

## 2. Methods

### 2.1. Preparation of Flaky CeO_2_ with Nanocrystals

The starting materials used were cerium nitrate hexahydrate (Ce (NO_3_)_3_·6H_2_O), aluminum nitrate nonahydrate (Al (NO_3_)_3_·9H_2_O), and ammonium bicarbonate (NH_4_HCO_3_), all of which were of analytically pure grade.

Firstly, a certain amount of Al (NO_3_)_3_ was dissolved in deionized water, and then Al (NO_3_)_3_ solution and NH_4_HCO_3_ solution as a precipitant were added in parallel flow. Magnetic stirring was used during the reaction. The reaction product was suction-filtrated, dried, and milled to prepare AlOOH.

As AlOOH is an amphoteric oxyhydroxide, it can react with an acid or a base. When the hydrolysis pH value is below 5 or above 10, some AlOOH precipitates will dissolve [12]. The AlOOH was dissolved in 1 mol/L Ce (NO_3_)_3_ solution at a pH of 4.3 in the reaction beaker, where the 2 mol/L NH_4_HCO_3_ solution as a precipitant was added drop by drop to raise the pH value to 7.5. At this stage, part of the AlOOH dissolved into Al^3+^, while the incompletely dissolved AlOOH helped with Ce^3+^ nucleation in the early stage of precipitation. After the reaction, the reaction product was aged for 24 h. The precipitates were filtered and dried at 120 °C for 2 h and calcined at different temperatures with a temperature increase rate of 10 °C/min. To investigate the effects of different calcination temperatures and Al contents, the samples were divided into groups numbered 1–4, consistent with 0:1, 1:5, 1:1, and 5:1 of the atomic ratios of Al:Ce, respectively. Then, the samples with 1:1 of Al:Ce were calcined at 350, 700, 900, and 1200 °C.

### 2.2. Chemical Mechanical Polishing (CMP)

To compare the polishing efficiency and effectiveness of the flaky CeO_2_ powder and commercial spherical CeO_2_ abrasives, the two abrasives were configured with Na_2_CO_3_ solution to form a simple polishing slurry of 3 wt.%. After thinning, SiC wafers were cut into 10 mm × 10 mm plates and polished with a flow rate of 10 mL/min and a rotational speed of 240 ppm for 1 h.

### 2.3. Characterization

Thermogravimetric Analysis (TG) and Differential Thermal Analysis (DTA) on a DTG-60H (Shimadzu, Shanghai, China) analyzed the precursors’ phase and mass changes with an increasing temperature. The phase analysis of the precursors and powders after calcination at different temperatures was carried out by X-ray diffraction (XRD) on a XRD6100 diffractometer (Shimadzu, Shanghai, China) with Cu Kα radiation (λ = 1.5418 Å); the XRD patterns were collected in the range of 2θ = 10°–90°. The changes in the chemical-bonding state of the elemental Ce due to changes in the ratio of Ce to Al in the CeO2 were investigated using X-ray photoelectron spectroscopy (XPS) on a Kratos AXIS Supra, with a monochromatic Al Kα X-ray source (hν = 1486.6 eV). All the binding energies were related to the C1s peak (284.8 eV).

The morphology of the samples was determined on a field emission scanning electron microscope (FESEM) Apreo S (Thermo Scientific™, Shanghai, China) and a transmission electron microscope (TEM) Tecnai G2 F20 S-TWIN (FEI, Shanghai, China).

The SiC surfaces before and after the polishing were compared to discuss the change in the surface. The surface profile and roughness were measured using a Contour GT-K optical profilometer from Bruker Nano Inc., Billerica, MA, USA.

## 3. Results and Discussion

### 3.1. Thermal Analysis and Composition

**Figure 1:**
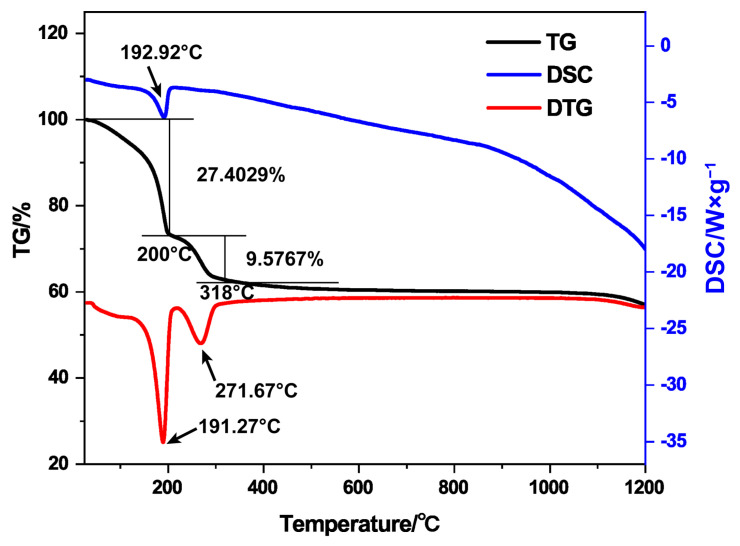
Thermal analysis curves of the precursors composed of CeOHCO_3_ and AlOOH.

As shown in Figure 1, the TG/DSC curves of the 6.5 mg precursors composed of CeOHCO_3_ and AlOOH ramping up at room temperature—1200 °C showed the first noticeable mass loss between 0 and 200 °C. The DSC curve showed a prominent heat absorption peak at 192.92 °C. In addition, the second more pr onounced mass loss occurred at 200–318 °C. The DTG curve showed a minimum at 191.27 °C, corresponding to the first mass loss and heat absorption. The minimum at 271.67 °C corresponds to the second weight loss. The TG/DSC curves showed that the precursor had a phase transformation when the calcination temperature was above 200 °C and other components changed during 200–318 °C.

**Figure 2:**
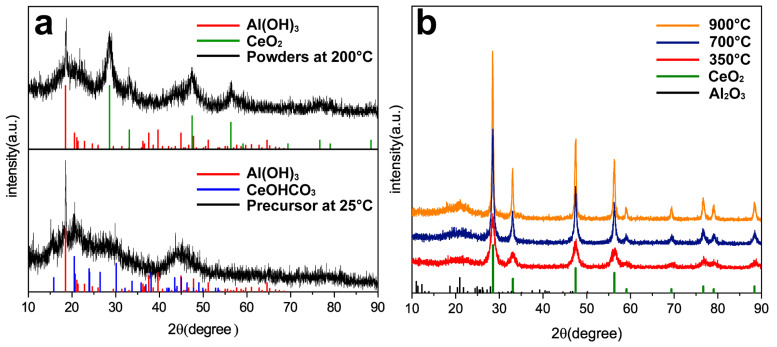
XRD patterns of (**a**) precursor and powder calcined at 200 °C, and (**b**) powder calcined at different temperatures.

The XRD patterns of the precursor prepared by ordinal precipitation and powder consisting of CeO_2_ and Al_2_O_3_ after calcination at different temperatures are shown in Figure 2. The precursor shows diffraction peaks of CeOHCO_3_ consistent with 41-0013 of the Joint Committee on Powder Diffraction Standards (JCPDS). This is because NH_4_HCO_3_, the precipitant, provides CO_3_^2−^ ions and creates an alkaline precipitation environment. In addition, there are diffraction peaks of Al(OH)_3_ consistent with 24-0006, which are from AlOOH combined with H_2_O [13].

CeO_2_ with a cubic fluorite structure is confirmed at 200 °C, and the diffraction peaks of the precursor CeOHCO_3_ are not observed. CeOHCO_3_ transforms into CeO_2_ according to Equation (1) when the temperature exceeds 200 °C [14], consistent with the thermal analysis.

CeOHCO_3_ + 1/4O_2_→CeO_2_ + 1/2H_2_O↑ + CO_2_↑ (1)

As the temperature increases to 350 °C, Al_2_O_3_ consistent with 31-0026 is confirmed and the diffraction peaks of Al(OH)_3_ cannot be observed, which means that Al(OH)_3_ is decomposed into Al_2_O_3_ and H_2_O according to Equation (2) [15]

2Al(OH)_3_→Al_2_O_3_ + 3H_2_O (2)

The theoretical quality change was calculated according to the inferred composition change. The weight loss curve of the first section with a large slope is related to the removal of free water and crystalline water from the sample. After a 10% weight loss, the precursor of 6.5 mg should contain CeOHCO_3_ weight 4.30 mg and Al(OH)_3_ weight 1.54 mg, with a molar ratio of 1:1. In the second stage of the weight loss, CeOHCO_3_ is converted into CeO_2_ and theoretically loses 0.90 mg (13.85%), while later Al(OH)_3_ is converted into Al_2_O_3_ and theoretically loses 0.19 mg (8.31%). The results of the theoretical calculation are consistent with the TG curve obtained in the experiment and confirm the composition change mechanism we inferred.

As the temperature increases to 700 °C and 900 °C, the diffraction peaks of CeO_2_ become sharper, implying better crystallinity. In terms of Scherrer’s formula, the average grain size of the sample can be calculated as 29.3 nm at 350 °C, 42.1 nm at 700 °C, and 49.5 nm at 900 °C based on the diffraction peak of about 28.5°.

### 3.2. Morphology and Microstructure

**Figure 3:**
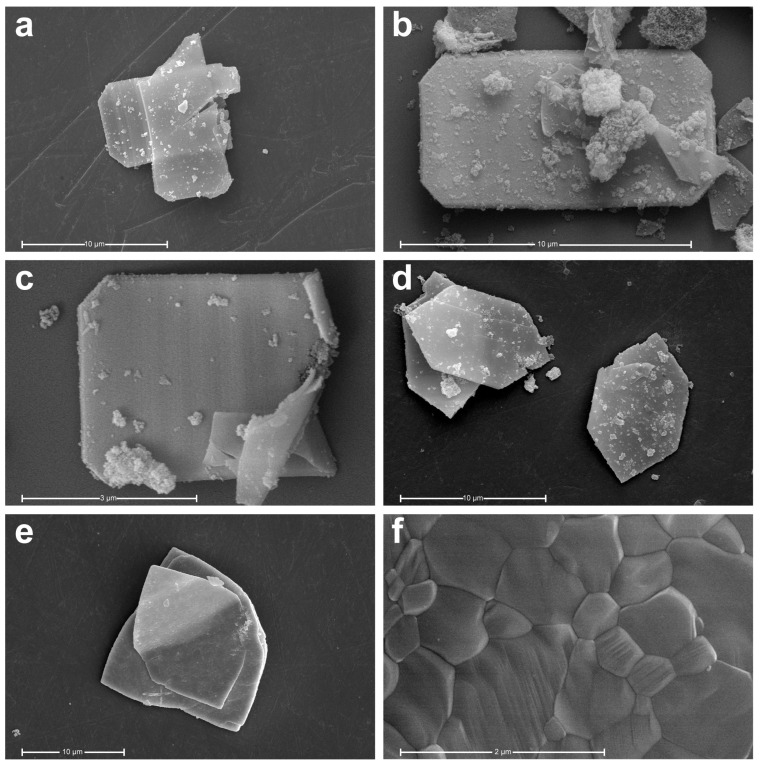
SEM images of the sample at different temperatures: (**a**) uncalcined precursor (**b**) 350 °C (**c**) 700 °C (**d**) 900 °C (**e**)1200 °C and (**f**) big grains at 1200 °C.

The excess Ce^3+^ in the reaction beaker was nucleated in large quantities using the ordinal precipitation method. Then, these fine grains were aggregated to form large flakes of material, as shown in Figure 3a.

The morphology of the samples calcined at different temperatures is shown in Figure 3b–d. All the powders are flaky-like, mainly octagonal when the temperature is below 900 °C, and grow to hexagonal at 900 °C. Some particles attached to the precursors and the powders after calcination.

The nanoparticles grow gradually to 400 nm for small grains and 1 μm for big grains when the temperature rises to 1200 °C, as shown in Figure 3e,f.

**Figure 4:**
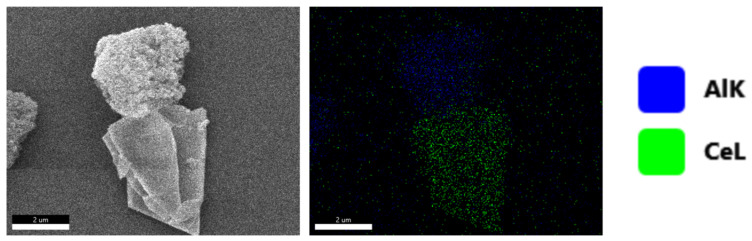
EDS images of the sample calcined at 900 °C.

To determine the morphological characteristics of the aluminum oxide and cerium oxide in the sample, EDS was used to detect the composition of the sample calcined at 900 °C. As shown in Figure 4, the morphology of the cerium oxide and aluminum oxide in the product is quite different. Cerium oxide is a large-sized flake material, while aluminum oxide is an agglomerate of nanoparticles.

**Figure 5:**
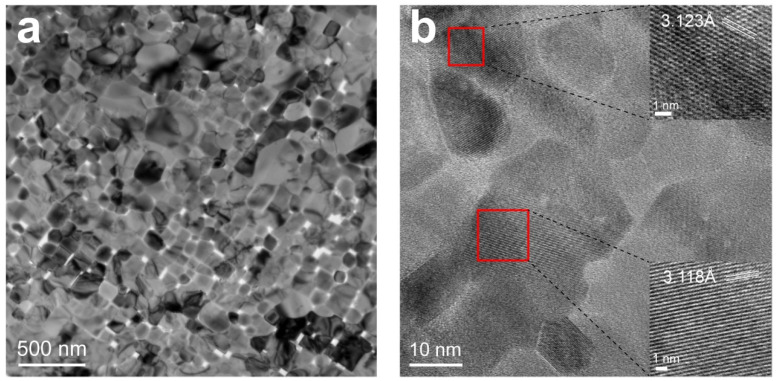
(**a**) TEM morphology of CeO_2_ grains and (**b**) lattice fringes of (111) in the HRTEM image.

To observe the nanocrystals in the flaky CeO_2_, a TEM was used to investigate the morphology of CeO_2_ calcined at 900 °C. The sizes of the nanocrystal grains in CeO_2_ calcined at 900 °C are about 50–150 nm (Figure 5a). It was accounted for that the precursor obtained using ordinal precipitation was composed of nano-sized grains. The nanograins in the flaky material are finer and have a uniform size distribution. The grains grew slowly as the temperature increased to 900 °C. Based on Figure 5b showing the HRTEM images, the highly crystallized cerium oxide nanograins have distinct lattice lines resolved at 3.123 and 3.118 Å, which belong to the (111) crystal planes of CeO_2_ crystals. Among several low-index lattice planes of CeO_2_, the (111) crystallographic plane is the one with higher stability [16], which explains that the principal crystallographic planes observed in the TEM images are (111) crystallographic planes.

**Figure 6:**
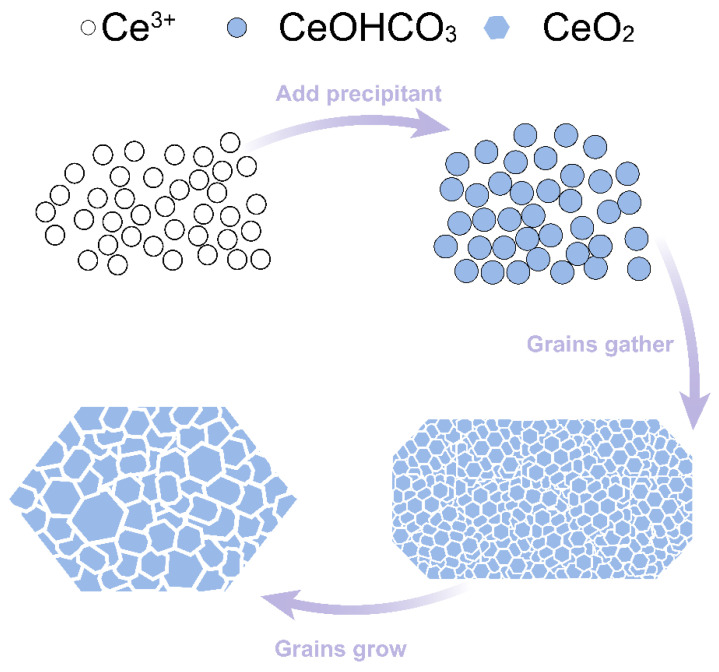
Illustration of the formation mechanism of flaky nanocrystal CeO_2_.

The growth mechanism is inferred from the morphological observation of the flaky nanocrystalline CeO_2_ material, as shown in Figure 6. As precipitant was added, Ce^3+^ rapidly nucleated into fine grains of CeOHCO_3_. At the same time, AlOOH acted as a template for the nucleation sites. AlOOH gradually dissolved into the cerium nitrate solution to release Al^3+^. Driven by the excellent surface energy, many fine grains were aggregated and formed flaky CeOHCO_3_. The pH value kept rising with the continuous addition of the precipitant, and AlOOH was transformed to Al(OH)_3_. Subsequently, during the calcination process, CeOHCO_3_ was converted into CeO_2_ with an increasing temperature under an atmosphere of air. Al(OH)_3_ transformed into Al_2_O_3_, which enhanced the mechanical strength of the abrasives. Tiny grains kept fusing and growing to become large with the increase in the temperature. Normally, CeO_2_ particles grow gradually as the temperature increases. However, the grains grow slowly as the temperature increases from 300 °C to about 900 °C. When the temperature increases to 1200 °C, the grains proliferate to the micrometer level. It can be considered that the slow diffusion of reactants limits the chemical reaction, meaning that the CeO_2_ grains grow in a small range by mass transfer [17].

### 3.3. Chemical State of Ce

**Figure 7:**
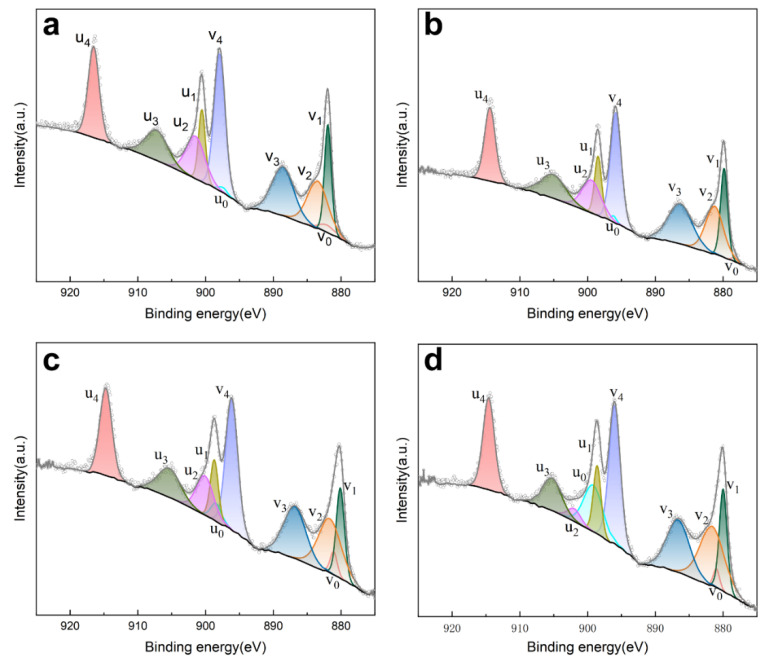
XPS spectra of CeO_2_ without AlOOH (**a**) and CeO_2_ with Al in ratio of (**b**) Al:Ce = 1:5 (**c**), Al:Ce = 1:1 and (**d**) Al:Ce = 5:1.

The Ce^3+^ in CeO_2_, rather than the Ce^4+^, is believed to improve the performance in polishing [7]. To investigate the effect of the content of the element Al on the chemical state of CeO_2_, samples 1–4 were quantitatively analyzed by X-ray photoelectron spectroscopy (XPS), as shown in Figure 7. Table 1 lists the detailed information of each XPS peak assignment. For the Ce 3d signal in the samples, it can fit the peaks of Ce 3d_5/2_ and Ce 3d_3/2_, and after the curve-fitting, the Ce 3d_2/5_ peaks were labeled as v_0_, v_1_, v_2_, v_3_, v_4_, and the Ce 3d_3/2_ peaks were labeled as u_0_, u_1_, u_2_, u_3_, u_4_. Moreover, v_0_, v_2_, u_0_, u_2_ belonged to Ce^3+^, while the v_1_, v_3_, v_4_, u_1_, u_3_, u_4_ peaks belonged to Ce^4+^ ions [18]. The Ce^3+^ content in the samples was calculated by measuring the area of each peak of the Ce^3+^ to the area of all the Ce 3d peaks. The Ce^3+^ content of the control group was 26.00%. When the content of Al was less, it had no obvious effect on the CeO_2_. With the gradual increase in the Al content at the ratio of Al:Ce of 1:1, the Ce^3+^ content reaches 28.48%. When the ratio of Al:Ce rises to 5:1, the Ce^3+^ content reaches 31.12% and has enhancement compared with that of the control group.

### 3.4. SiC Surface Polished by CeO_2_

**Figure 8:**
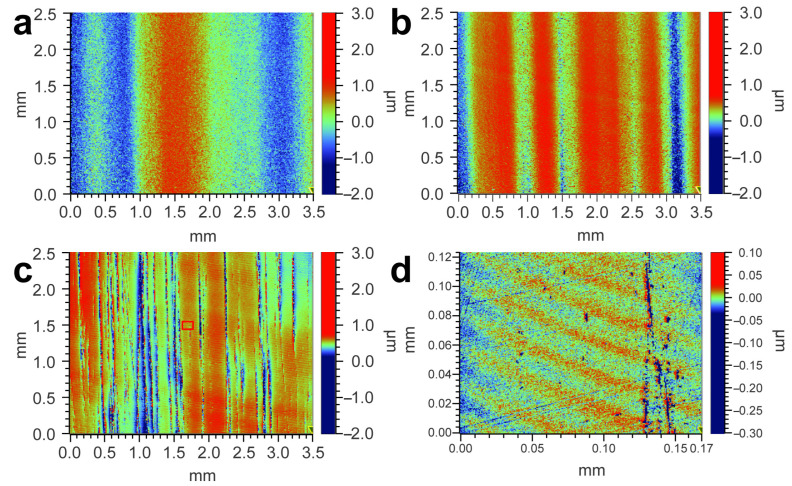
Surface profile of (**a**) SiC before polishing, (**b**) polished by common spherical CeO_2_ abrasive, (**c**) polished by flaky CeO_2_ abrasive, and (**d**) microregion of SiC polished by flaky abrasive.

Powder consisting of flaky CeO_2_ with Al_2_O_3_ (about 10 μm, calcined at 900 °C and added AlOOH in the ratio Ce:Al = 1:5) and common spherical CeO_2_ abrasive (2.3 μm) were used to polish SiC wafers to compare the polishing performance. In Figure 8a, the SiC surface before polishing exhibits surface characteristics of alternating high and low elongated bars, and the surface roughness Ra is 464 nm. In Figure 8b, the height differences of the surface polished by spherical CeO_2_ abrasive are reduced, and the roughness Ra is reduced from 464 nm to 230 nm. In Figure 8c, the height differences of the surface polished by flaky CeO_2_ are the lowest, with a Ra roughness of 67 nm. Figure 8d shows the optical profile of a local higher area of 0.17 mm × 0.12 mm in the red box of Figure 8c, and the Ra is measured as 11 nm. The surface roughness and the height difference are much lower than those associated with polishing by spherical abrasives.

## 4. Conclusions

In conclusion, a novel flaky CeO_2_ composed of nanocrystals was obtained using ordinal precipitation. AlOOH was added to contribute to the nucleation of the nanograins. Meanwhile, Al^3+^ doped in CeO_2_ facilitated the increase in Ce^3+^, which was proved by XPS analysis. When the Al^3+^ was doped in the ratio Al:Ce = 5:1, the Ce^3+^ increased from 26.00% to 31.11%. Al^3+^ without doping in CeO_2_ transformed into Al_2_O_3_, enhancing the mechanical strength of the powder. After the SiC wafer was polished by a slurry containing flaky CeO_2_ for 1 h, the height differences decreased significantly, and the surface roughness Ra was reduced from 464 nm to 11 nm in an area of 0.17 mm × 0.12 mm.

## Figures and Tables

**Table 1:** XPS peak assignments of Ce 3d.

No.	Ce 3d_5/2_					Ce 3d_3/2_					Ce^3+^ (%)
	v0	v1	v2	v3	v4	u0	u1	u2	u3	u4	
	Ce^3+^	Ce^4+^	Ce^3+^	Ce^4+^	Ce^4+^	Ce^3+^	Ce^4+^	Ce^3+^	Ce^4+^	Ce^4+^	
1	1.56	10.07	13.14	14.47	20.3	0.61	6.67	10.69	9.02	13.6	26.00
2	0.39	10.32	13.89	15.77	19.63	0.59	6.84	10.09	9.45	13.02	24.96
3	2.05	9.26	15.63	14.62	20.02	2.41	6.14	8.39	8.2	13.27	28.48
4	1.37	9.39	17.23	14.32	18.96	10.51	6.22	2.01	7.41	12.57	31.11

## Data Availability

Data are contained within the article.
